# Exploring larger evidence base for contemporary Ayurveda

**DOI:** 10.4103/0974-7788.72497

**Published:** 2010

**Authors:** Sanjeev Rastogi

**Affiliations:** *Department of Kaya Chikitsa, State Ayurvedic College, Tulsi Das Marg, Lucknow - 226 003, India E-mail: rastogisanjeev@rediffmail.com*

Sir,

“Exploring larger evidence-base for contemporary Ayurveda”, a guest editorial by Prof. Singh in IJAR April 2010 issue,[1] gives us an opportunity to rethink how the classical frame of evidence from Ayurveda can be utilized to a contemporary tune. While talking of searching and applying evidence base to Ayurveda, we need to understand that in conventional sense, the term is primarily coined to help in clinical decision making in the light of best among overwhelmingly available evidences within modern medicine.[2] Conventional modus operandi of applying evidence base to medical practice therefore is limited to screening of best evidences that help in decision making. Thereby, defining the best evidence and finding them pragmatically in a clinical setting are the only real challenges.

Referring to Ayurveda, however, the issue of applying evidence base needs to be redefined in reference to its unique propositions. There is a ubiquitous agreement upon the traditional evidences of Ayurveda of which experience, long-term use, and textual classical references form a large sum. This substantial evidence base of Ayurveda, however, is required to be brought in a format which can become retrievable to help with decision making in a clinical dilemma. A thorough documentation therefore comes as the foundation of evidence-based practice and it warrants an unfailing and untiring documentation of every evidence from Ayurveda, conventional or unconventional.[3] Besides, this is also important to address the issue of human biology as is observed from ayurvedic or conventional perspective. If we are deferring from conventional theories and their experimental designs, we need to innovate our own methods to understand a biological process and also the ways through which this understanding can help with decision making.[4] Needless to emphasize, these methods are essentially required to be flawless, dependable, reproducible and acceptable. Designing these methods requires a rigorous brainstorming initially about what is available and what is needed, and subsequently, the ground research by designing suitable models which can solve the dilemma of understanding the ayurvedic biology.[5]

The issue of evidence base in Ayurveda therefore requires to be dealt at various levels like documentation of existing evidences, designing diagnostic and clinical parameters which can act as evidence to help in decision making and generating more evidences in reference to the safety and efficacy pertinent to ayurvedic practice. This is the time when we need to understand that bringing evidence base to the practice of Ayurveda is mandatory if it is thought to be raised as a medical system where predictability and dependability are featured as key components.
